# A Rare Case of Type II Adult Hypertrophic Pyloric Stenosis Secondary to Chronic Peptic Ulcer Disease

**DOI:** 10.7759/cureus.85037

**Published:** 2025-05-29

**Authors:** Riah S Lee, Sylvia Dygulski, Yasmine Hemida, Guillermo M Uy

**Affiliations:** 1 Surgery, Touro College of Osteopathic Medicine, Middletown, USA; 2 General Surgery, Garnet Health Medical Center, Middletown, USA; 3 General Surgery, Crystal Run Healthcare, Middletown, USA

**Keywords:** adult hypertrophic pyloric stenosis, esophagogastroduodenoscopy (egd), gastrectomy, gastric outlet obstruction (goo), pyloric stenosis

## Abstract

Adult hypertrophic pyloric stenosis (AHPS) is a rare subtype of gastric outlet obstruction (GOO) that occurs without a history of recurrent vomiting or other gastrointestinal symptoms in infancy. AHPS type 2 is the most common type of AHPS that arises from underlying GI pathologies such as recurrent peptic ulcer disease (PUD), malignancy, vagal hyperactivity, and extrinsic adhesions.

A 43-year-old male with no history of vomiting in infancy presented with a two-day history of intractable nausea, nonbilious and non-bloody forceful vomiting, globus pharyngeus, and worsening dysphagia to both solids and liquids. The patient had experienced similar episodes in the past, all of which were managed conservatively. The computed tomography (CT) of the abdomen/pelvis with IV contrast showed marked distention of the stomach, unable to rule out GOO. Esophagogastroduodenoscopy (EGD) revealed multiple clean-based ulcers with a pinhole-sized opening at the pylorus, making it difficult to pass. A robotic distal gastrectomy with Roux-en-Y reconstruction was subsequently performed without major complications. The pathological examination of the specimen showed focal, moderate, nonspecific chronic inflammation but was negative for *Helicobacter pylori*, acute inflammation, granuloma, dysplasia, or tumor. The recovery was uneventful, and the patient was discharged without any complications.

With history, physical examination, and radiography often failing to provide definitive results, fiberoptic gastroscopy with histopathological examination is a more reliable diagnostic tool. While nonsurgical management, such as proton-pump inhibitors, has shown mild efficacy in treatment, partial gastrectomy remains the definitive surgical approach for symptomatic AHPS.

AHPS is a rare cause of gastric outlet obstruction in adults, and diagnosis is based upon radiological and endoscopic findings after excluding other common causes of GOO. We emphasize the importance of including AHPS in the differential diagnosis for patients presenting with globus pharyngeus and dysphagia.

## Introduction

Hypertrophic pyloric stenosis primarily affects infants, but in rare cases, it can occur in adults as adult hypertrophic pyloric stenosis (AHPS) [1]. Since the first discovery of the pathological anatomy by Jean Cruveilhier in 1842, roughly 200 cases have been documented [1,2]. Along with its infrequency, AHPS poses a diagnostic challenge due to its nonspecific imaging findings along with variable symptomatic presentations. The most common symptoms include insidious nausea and nonbilious, projectile vomiting, early satiety, abdominal distension, and epigastric pain [1]. While AHPS is a benign disease, its rarity limits proper diagnosis in adults and is often confused with malignant or benign tumors [3]. Despite being exceedingly uncommon, this diagnosis should not be overlooked in the differential, as early identification can significantly improve quality of life and clinical outcomes. We hope to increase awareness and timely recognition of this diagnosis by reporting a case of AHPS type II presenting with globus pharyngeus and dysphagia secondary to chronic peptic ulcer disease (PUD).

## Case presentation

**Figure 1:**
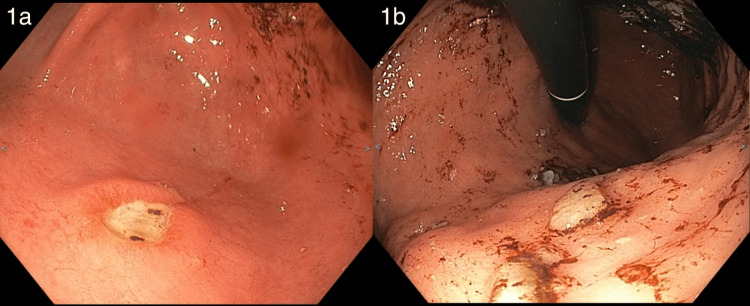
(a) An elevated gastric ulcer with exudate at the base. (b) Multiple 4-8 mm clean-based gastric ulcers.

**Figure 2:**
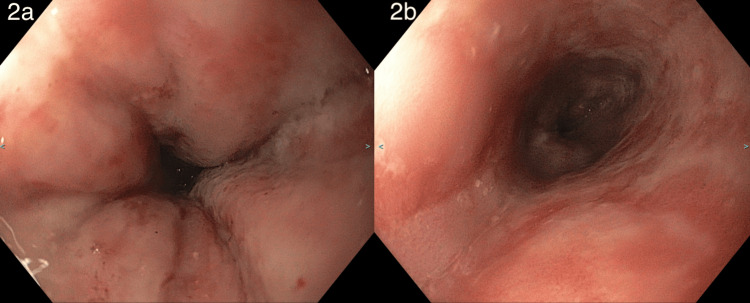
(a) A pinhole-sized narrowing at the pylorus, scoping being unable to pass. (b) Pyloric edema and multiple ulcerations.

This patient is a 43-year-old male with a history of gastric outlet obstruction (GOO), esophagitis, gastroesophageal reflux disease (GERD) with *Helicobacter pylori*, PUD, and dysphagia and presented to the emergency department multiple times with a two-day history of intractable vomiting. During the first visit, CT imaging of the abdomen and pelvis showed esophagitis and a distended stomach, which was concerning for gastritis, gastroparesis, and gastric outlet obstruction (GOO). Esophagogastroduodenoscopy (EGD) showed severe gastric ulcers and ulcerative esophagitis (Figure 1, Panels a and b). The duodenum could not be observed due to a pinhole-sized opening at the pylorus (Figure 2, Panels a and b). The patient recovered after conservative measures, including intravenous (IV) proton-pump inhibitor (PPI) and bowel rest, and was subsequently discharged.

Despite initial clinical improvement, the patient returned in three days with worsening nausea and vomiting. Another EGD was performed, which showed distal esophagitis and pyloric stenosis with edema. The location of the Z-line could not be determined due to esophagitis. Similar to the first EGD, the scope was still unable to pass the pylorus. The biopsied specimen showed squamous and cardiac gastric mucosa with nonspecific chronic inflammation. H. pylori testing was negative with no dysplasia or malignancy found. Different treatment options were considered, but pyloric dilation was not recommended due to an increased risk of perforation secondary to severe ulcers. The patient recovered after conservative measures and was subsequently discharged with PPIs and sucralfate, with an elective partial gastrectomy scheduled within a month.

The patient returned two weeks later with worsening symptoms of nausea, vomiting, globus sensation, and dysphagia to both solids and liquids. The patient was afebrile with stable vital signs but had a mild leukocytosis of 12,500/µL (Table 1).

**Table 1:** Patient vitals with reference ranges

	Patient Vitals	Reference Range
Blood pressure	103/62	Systolic: 90–120 mmHg; diastolic: 60–80 mmHg
Heart rate	66	60–100 bpm
Respiratory rate	18	12–20 breaths/min
Temperature	97.9 (oral)	97.0°F–99.0°F (oral)
SpO_2_	97%	95%-100%

**Figure 3:**
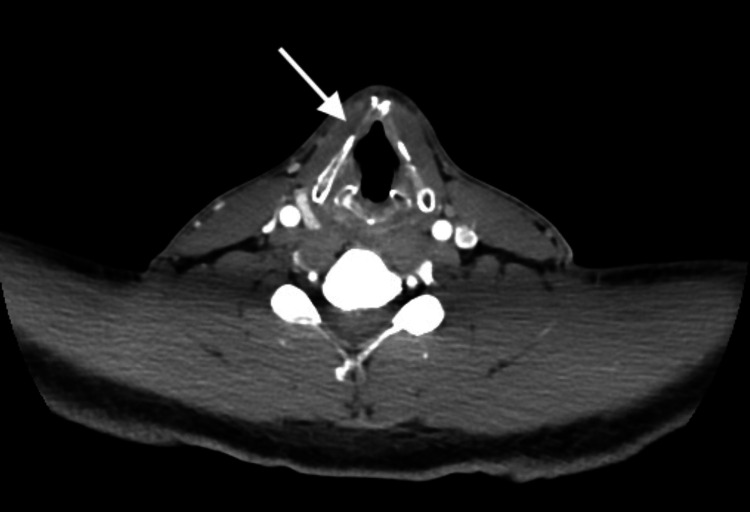
CT of the neck with contrast showing right vocal cord medialization

Interestingly, the physical examination was unremarkable except for the patient’s limited ability to speak normally. The CT of the neck with contrast subsequently showed medialization of the right vocal cord (Figure 3). The CT of the abdomen/pelvis with IV contrast showed marked distension of the stomach, unable to rule out GOO.

**Figure 4:**
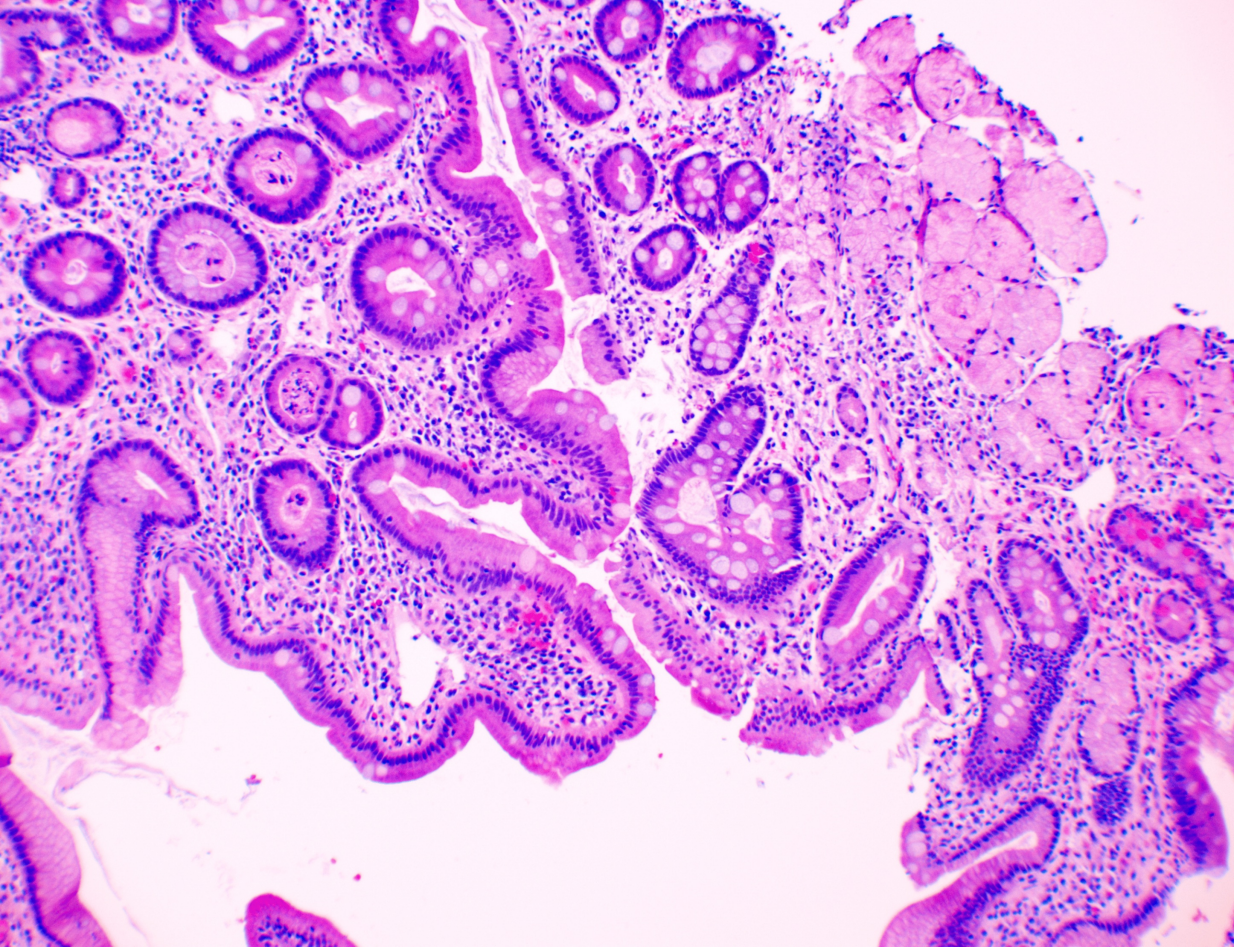
Pyloric mucosa with superficial mild nonspecific chronic inflammation and intestinal metaplasia (H&E stain, 40x)

An elective robotic distal gastrectomy with Roux-en-Y reconstruction was performed. Intraoperatively, the duodenal bulb was noted to have hypertrophic scarring at the posterior aspect. Histopathological examination of the specimen revealed pyloric mucosa with superficial mild nonspecific chronic inflammation (Figure 4). A thickened muscularis propria was also noted along the pylorus without significant pathologic change, confirming the diagnosis of AHPS.

**Figure 5:**
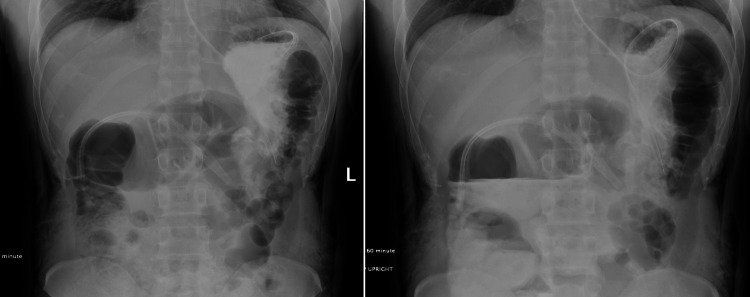
Panel A (left) shows minimal contrast in the stomach within the first five minutes, while Panel B (right) demonstrates the gradual passage of contrast from the stomach through the gastrojejunostomy into the intestine without leakage after one hour

The upper gastrointestinal (UGI) series was performed on postoperative day 2 (POD). Results from the UGI series showed postoperative changes consistent with a gastrojejunostomy, with a delay in the Gastrografin leaving the stomach through the gastrojejunostomy. During the first five minutes of the study, almost no contrast left the stomach (Figure 5, Panel A). However, over the next hour, there was a gradual exit of the contrast from the stomach through the gastrojejunostomy into the intestine (Figure 5, Panel B). There was no obstruction or extravasation from gastrojejunostomy. The recovery was uneventful, and the patient was subsequently discharged on POD 4 with instructions to follow up. The patient was also referred to an ENT specialist for further assessment of the medialization of the vocal cord. To date, the patient has no residual symptoms of nausea, vomiting, dysphagia, or globus pharyngeus.

## Discussion

AHPS is a rare disease with almost 200-300 cases reported in the literature, which most often presents as a pyloric obstruction [1]. Since the first pathological discovery by Cruveilhier in 1842, hypertrophic pyloric stenosis has been classified in multiple ways by various authors [2]. The most commonly accepted classification was first proposed by Danikas et al., who categorized HPS into three distinct types [4]. Type I, or the idiopathic form, is found in infancy but is undiagnosed until later in adulthood, whereas type II is found in adults but is formed secondary to other upper gastrointestinal tract diseases such as peptic ulcers, malignancy, or inflammatory diseases. Lastly, type III is idiopathic without an apparent underlying cause [4]. The second type is by far the most prevalent form and is also the type that was observed in our patient. Males are more likely to be affected, and it is more commonly diagnosed in the fourth and fifth decades of life [5].

Patients with AHPS present with early satiety, abdominal distention, nausea, vomiting, abdominal pain, and eructation [6]. Our patient presented with similar symptoms but also with a worsening globus sensation. Globus pharyngeus is a non-painful sensation of a foreign body in the throat and has an uncertain etiology. GERD has been commonly associated with globus pharyngeus, with a study citing 38.7% of patients with GERD having symptoms of globus pharyngeus [7]. Mechanisms of GERD have been proposed to explain the globus sensation, including direct irritation and inflammation of the laryngopharynx due to retrograde flow of gastric contents and vagovagal reflex hypertonicity of the upper esophageal sphincter (UES) triggered by acidification or distention of the distal esophagus [8]. While a direct association between globus pharyngeus and AHPS has not been reported, this is most likely secondary to gastroesophageal reflux disease and chronic PUD [9].

Imaging findings such as a concentric narrowing of the pylorus (string sign) or convex indentation at the duodenal bulb (Kirklin sign) may be suggestive of AHPS, although they are highly variable [5]. Endoscopic findings include the narrowing of the pylorus, also known as the cervix sign [10]. However, these examinations can often be normal, making it more difficult to diagnose with imaging [11]. Abdominal CTs can be helpful to exclude secondary types of AHPS but will often be nonspecific. Similarly, endoscopy can also show narrowing of the pylorus, just as in our patient’s case. However, pathological examination is warranted for a definitive diagnosis [12]. Pathological specimens can show grossly elongated or thickened pylorus along with microscopic hypertrophy and hyperplasia of the gastric muscularis propria [10].

With outlet obstruction, a gastric scintigraphy may show delayed emptying, which can lead to a misdiagnosis of gastroparesis [13]. Other differential diagnoses include malignancy, PUD, and gastrointestinal stromal tumors (GISTs). Treatment options include gastrojejunostomy, gastrectomy, pyloroplasty, vagotomy, pyloromyotomy, and endoscopic balloon dilation, although pyloroplasty and vagotomy have higher recurrence rates [14,15]. Currently, there is no evidence of one surgical technique being superior to the other [1,16]. However, distal gastrectomy with Billroth I is the most commonly performed surgical approach for AHPS [16]. After discussion with the primary care team, gastroenterology, and the patient, distal gastrectomy with Roux-en-Y was performed for more permanent treatment of AHPS. Given the patient’s extensive medical history involving recurrent gastritis, GERD, and PUD, distal gastrectomy with Roux-en-Y was performed [17,18].

## Conclusions

AHPS is a rare cause of GOO in adults. Its diagnosis is challenging due to nonspecific symptoms, variable image findings, and clinical overlap with other obstructive pathologies. Histopathologic evaluation remains the gold standard for diagnosis. Underlying GI conditions, such as recurrent PUD, can predispose a patient to AHPS. Conservative treatment as well as partial gastrectomy is recommended for optimal results to effectively address the obstruction while preserving the function of the stomach and duodenum. Continued follow-up is important to monitor for complications such as anastomotic leakage or symptom recurrence. This case underscores the importance of considering AHPS in adults presenting with similar symptoms and reinforces the role of a meticulous workup in determining optimal management.
